# Priming determinist beliefs diminishes implicit (but not explicit) components of self-agency

**DOI:** 10.3389/fpsyg.2014.01483

**Published:** 2014-12-17

**Authors:** Margaret T. Lynn, Paul S. Muhle-Karbe, Henk Aarts, Marcel Brass

**Affiliations:** ^1^Department of Experimental Psychology, Ghent UniversityGhent, Belgium; ^2^Department of Psychology, Utrecht UniversityUtrecht, Netherlands

**Keywords:** free will, agency, self-control, unconscious, intentional binding

## Abstract

Weakening belief in the concept of free will yields pronounced effects upon social behavior, typically promoting selfish and aggressive over pro-social and helping tendencies. Belief manipulations have furthermore been shown to modulate basic and unconscious processes involved in motor control and self-regulation. Yet, to date, it remains unclear how high-level beliefs can impact such a wide range of behaviors. Here, we tested the hypothesis that priming disbelief in free will diminishes the sense of agency, i.e., the intrinsic sensation of being in control of one’s own actions. To this end, we measured participants’ implicit and explicit self-agency under both anti-free will and control conditions. Priming disbelief in free will reduced implicit but not explicit components of agency. These findings suggest that free will beliefs have a causal impact on the pre-reflective feeling of being in control of one’s actions, and solidify previous proposals that implicit and explicit agency components tap into distinct facets of action awareness.

## INTRODUCTION

The question of whether free will truly exists has fascinated philosophers, psychologists, and neuroscientists for centuries (e.g., Libet et al., 1983; Wegner, 2004; Haggard, 2008). Yet contemporary empirical research typically avoids the existential question itself, and instead focuses on more tangible research questions concerning the consequences of (dis)belief in free will, and its relation to agentic causation and volition.

Seminal studies in the domain of social psychology have shown that weakening belief in the concept of free will, via reading of essays or statements that promote a determinist perspective, seems to be capable of impacting participants’ subsequent social behavior. For instance, Vohs and Schooler (2008) found that participants who were primed with disbelief in free will paid themselves a statistically unlikely and disproportionally large amount of money, and took advantage of opportunities to cheat more often than a group of control participants who read texts unrelated to free will. Likewise, Baumeister et al. (2009) found that a similar manipulation was able to increase participants’ aggression and decrease their helping behavior. These findings indicate that free will beliefs might be crucial for maintaining the motivation necessary to control selfish impulses in favor of pro-social behavior, in accordance with societal norms. Note, however, that there is a considerable controversy in the literature about the impact of free-will beliefs on behavioral control (e.g., Miles, 2013) and recent evidence indicates that believing in determinism may also have arguably positive side effects such as reduced retributive attitudes (see Shariff et al., 2014).

More recent work in the field of experimental psychology has revealed that the effects of weakening participants’ free will beliefs are not restricted to complex social behavior, but even propagate to very basic levels of motor control (Rigoni et al., 2011, 2013; Lynn et al., 2013). Using a similar procedure to that of Vohs and Schooler (2008), Rigoni et al. (2011) found that inducing disbelief in free will was associated with a reduced amplitude of the readiness potential, an electrophysiological marker of pre-conscious movement preparation (Libet et al., 1983). In follow-up studies, it was found that weakening free will beliefs also influenced the effectiveness of other basic adaptive control processes, such as post-error slowing (Rigoni et al., 2013) or the intentional inhibition of pain avoidance behavior (Lynn et al., 2013). This suggests that weakening free will beliefs counteracts the recruitment of self-regulatory resources to adapt behavior in response to environmental demands.

However, despite these recent advances in research on the impact of free will beliefs on behavioral control, the mechanisms underlying the crosstalk between high-level beliefs and low-level sensorimotor processes remain poorly understood. To explain their original finding regarding the readiness potential, Rigoni et al. (2011) speculated that weakening free will beliefs may reduce participants’ *sense of agency*, i.e., the intrinsic experience of being in control of one’s own actions (for reviews see Gallagher, 2000; Haggard and Chambon, 2012). This altered experience may then, in turn, hamper the recruitment of intentional effort for action production. Yet, despite the principle plausibility of this view, to date there exists only preliminary correlational supporting evidence.

Building on the notion that belief in free will often co-occurs with the pursuit of goal-directed behavior, Aarts and van den Bos (2011) tested the possibility that free will beliefs are associated with implicit processing of action-outcome relations underlying goal-directed behavior. The authors compared participants with either strong or weak dispositional free will beliefs in two different tasks that tapped into implicit aspects of agentic experience: (1) an intentional binding task, which measures the perceptual attraction of an intentional action and its sensory outcomes in terms of time, and (2) an action-outcome priming task, which assesses agency inferences resulting from a match between primed and actual outcomes (see Moore and Obhi, 2012; van der Weiden et al., 2013; for reviews). Aarts and van den Bos (2011) found that strong dispositional free will beliefs were associated with greater intentional binding and a stronger influence of primes on agency inferences. These, along with related findings (Desantis et al., 2011; Dogge et al., 2012), clearly indicate that free will beliefs and agency are related. In particular, they suggest that free will beliefs modulate the strength of predictive signals about action-outcomes. Nevertheless, given the correlational nature of the above study, a causal link between the two concepts remains to be established. Accordingly, the primary goal of the present study was to scrutinize the hypothesis that free will beliefs have a direct and causal impact on the sense of agency. Such evidence would provide the missing link to explain previous findings employing anti-free will manipulations, and highlight a general mechanism through which beliefs can affect even basic and unconscious adaptive processes. To this end, we employed the same procedure to manipulate the strength of free will beliefs as used in previous studies (e.g., Vohs and Schooler, 2008) in a within-subjects design. Participants were invited for two visits, in which they read essays that promoted either disbelief in free will or outlined general statements about consciousness (serving as a control condition).

Our secondary goal was to specify which aspects of agency are related to free will beliefs. It has been argued that the sense of agency constitutes a multi-dimensional construct comprising both an *implicit*, pre-reflective, or non-conceptual component that is related to lower-level perceptual and motor experiences, and an *explicit*, reflective or conceptual component that is related to higher-level thoughts and attributions (Synofzik et al., 2008). The findings by Aarts and van den Bos (2011) indicate that free will beliefs are related to implicit processes, yet, so far, it is unclear to what extent their influence may propagate to the conscious, deliberative level of explicit agency. Thus, in order to measure the effects of the induction procedure on participants’ agency, we used two different experimental paradigms. To assess implicit components of agency, we employed an intentional binding task (see above and Materials and Methods). To assess explicit components of agency we used a modified version of the action-outcome learning method introduced by Sato and Yasuda (2005). This task requires participants to explicitly learn action-outcome relations and to subsequently rate their perceived agency over the outcomes. The benefit of this latter task is that it is explicit at all levels of processing; participants have full awareness of their actions and the corresponding agency cues (see Meterials and Methods section for a comprehensive description). Comparing the effects of inducing disbelief in free will in these two tasks allowed us to test our hypothesis that free will beliefs have a causal impact on the sense of agency, for both implicit and explicit components of agency.

## MATERIALS AND METHODS

### PARTICIPANTS

Fifty-two students of Ghent University (aged 18–24; 40 males) received a compensation of 16€ for their participation. The study was conducted in accordance with the Declaration of Helsinki, and the approval of Ghent University’s Ethical Committee was obtained in advance. All participants reported being naïve as to the purpose of the experiment. One participant was excluded in advance of analysis for failing to return for their second session.

### PROCEDURE

Participants were individually tested during two sessions, taking place on the same weekday and time over two consecutive weeks. Each participant completed one ‘*control*’ and one ‘*anti-free will*’ session, with session order counterbalanced across participants. Each session began with participants reading one of two possible essays by Francis Crick, “The General Nature of Consciousness” or “A Postscript on Free Will” (Crick, 1995, pp. 13 and 265 respectively). To ensure a thorough reading of the material and obfuscate the goal of the experiment, participants were informed that they would be tested regarding the material at two points during the session, and that memory retention was a major outcome of interest. In the control session, the excerpt was a brief historical overview of consciousness research, while the excerpt read during the anti-free will session questioned the reality of free will and posited that such a notion was pre-scientific (see Vohs and Schooler, 2008 for a similar procedure). Participants were invited to take as much time as they pleased to read and review the texts. Following the readings, participants were asked to complete the first of two tasks (either the intentional binding task or the ‘Sato’ task, see below), the order of which was counterbalanced across participants. Subsequent to the first task, participants were asked to write a brief essay summarizing the previously read excerpt. Participants were then given the chance to reread the text prior to commencing the second task. After the completion of both tasks, participants were asked to fill out four questionnaires in their first session (the FAD+, the BIS/BAS, the LOC, and the PANAS-X) and two in the second session (the FAD+ and PANAS-X). The FAD+ (Paulhus and Carey, 2011) is a 27-item inventory that measures free will and deterministic beliefs, and served as the basis for our manipulation check (e.g., “People must take full responsibility for any bad choices they make”). The PANAS-X (Watson et al., 1988) measures current mood. The BIS/BAS (Carver and White, 1994) is a measure of both behavioral inhibition and behavioral activation (cf. Gray, 1982), while the LOC (Rotter, 1966) measures a participant’s locus of control. The latter questionnaires served exclusively as fillers to shield participants from discovering the purpose of our experiment following the first session. Once participants had completed the questionnaires they received a written quiz on the essay, and in their second session, a short debriefing. The quiz was not scored but only served to reinforce our cover story. The debriefing questionnaire consisted of general questions regarding the experiment: how participants felt about the duration of the experiment, whether they felt their concentration slip, whether they had used any specific strategies, and finally, what they thought the purpose of the experiment was.

### MANIPULATION CHECK

To probe the general effectiveness of the manipulation in reducing participants’ free will beliefs, we compared their scores on the free will subscale of the FAD+ following the control and AFW sessions via paired samples *t*-tests (*p* = 0.05, one-tailed ^1^). Moreover, we computed a session-based difference score on the free will subscale as an individual screening of a given participant’s response to the manipulation. In previous studies, we observed that a small but meaningful number of participants respond to the AFW manipulation in a reactant way, i.e., they reported a *stronger* belief in free will after reading an anti-free will text. Accordingly, we also wanted to explore how a reactant response affects the resulting level of agency. To this end, we conducted (explorative) *post hoc* analyses in which the data of responders (i.e., participants whose free will beliefs were weakened in the AFW session) and reactant participants (i.e., participants whose free will beliefs were augmented in the AFW session) were analyzed separately.

### INTENTIONAL BINDING TASK

**FIGURE 1:**
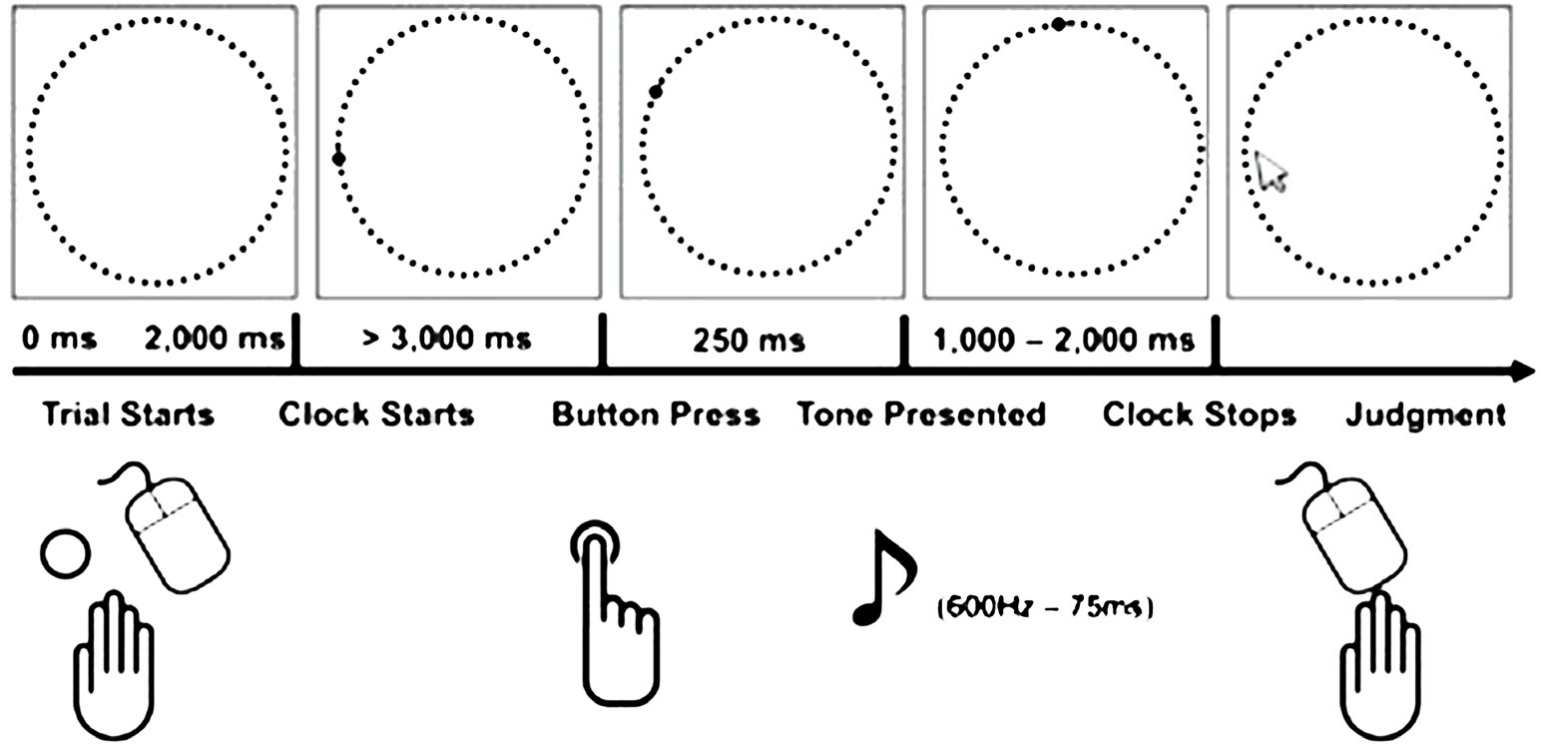
**Illustration of the single-trial structure of the intentional binding task (adapted from Demanet et al., 2013)**.

As a measure of implicit agency components, we used a modified version of the ‘intentional binding’ method introduced by Haggard et al. (2002a,b). Intentional binding refers to the temporal attraction of an action and its sensory effects within the actor’s perception (see Moore and Obhi, 2012, for a review). In this task, participants made time judgments about either actions or sensory events (tones) while these events occurred together or in isolation. They attended to the image of a centrally presented circular clock face (diameter = 8 cm) consisting of 60 dots (diameter = 2 mm). On every trial, a circular clock hand (diameter = 4 mm) rotated clockwise along the dots at a rate of 3 s per rotation, starting from an unpredictable clock hand position. Four different block types were employed, differing with respect to the event to be judged (action vs. tone) and whether or not there was an instrumental relation between actions and tones (agency vs. baseline). In the agency conditions, participants were instructed to press a response key (keyboard space bar) with their right hand at a moment of their choosing. Their responses were followed by a brief sine wave tone (frequency = 600 Hz, duration = 75 ms) presented via headphones at a delay of 250 ms, while the clock hand continued rotating for an unpredictable interval (varied between 1000 and 2000 ms in steps of 250 ms) and then disappeared. After the clock hand disappeared, participants were prompted to indicate the perceived time of either their button press (agency action) or the tone onset (agency tone) by manually selecting (mouse clicks) the corresponding clock hand position. In the two baseline conditions, temporal judgments were made about actions (baseline action) or about tones (baseline tone) when these events occurred in isolation. Participants performed 20 trials for each block type, resulting in 80 trials overall. The block order was counterbalanced across subjects. To estimate individual intentional binding scores, we first calculated the mean judgment errors (i.e., the deviance between perceived vs. actual time points of either actions or tones) for each block type and experimental session. Thereafter, binding scores were calculated for actions and for tones by subtracting judgment errors in the agency blocks from those in the corresponding baseline blocks. Finally, the overall amount of intentional binding was computed by adding the absolute values of both binding scores (see **Figure 1**, for a graphical illustration of the task). Mean judgment errors were analyzed in a general linear model (GLM) with the within-subjects factors JUDGMENT (action vs. tone), AGENCY (baseline vs. agency) and SESSION (control vs. anti-free will). To test whether intentional binding was modulated by the free will manipulation, we conducted *post hoc* paired-samples *t*-tests between the overall intentional binding scores in the control session and the anti-free will session (one-tailed; *p* < 0.05).

### SATO TASK

To assess explicit components of agency, we employed an adapted version of the task introduced by Sato and Yasuda (2005). The rationale of this task is grounded in ideomotor theory (e.g., Prinz, 1997; Elsner and Hommel, 2001), and the task entails two distinct experimental phases. In an initial *acquisition phase*, novel action-effect associations are explicitly formed by means of frequent and contingent pairing of simple responses and resulting sensory outcomes. In a subsequent *test phase*, participants perform the same task and judge their perceived agency over the action-outcome, while both the congruency of the action effect (compared to the acquisition phase) and its delay are manipulated.

In the acquisition phase, trials started with the presentation of the outline of a white square in the center of the screen. Participants were instructed to press either the NUM-del (with their right index finger) or NUM-enter button (with their right middle finger). Immediately after a response was given, the white square was replaced by a colored square with a specific color being assigned to each response (red and blue in one session, and yellow and green in the other; sequence of color sets counterbalanced across subjects). Participants were instructed to freely choose their responses in each trial, but to try to achieve an equal overall frequency of both responses without using a particular strategy such as simple alternations between left and right responses. Altogether, the acquisition phase comprised 200 trials, a number of repetitions that has been shown to be sufficient to establish strong action-effect representations (see Elsner and Hommel, 2001).

In the test phase, participants were told that on some trials their action would cause the colored square to appear on the screen, while on other trials the computer would cause the colored square, and their task would be to infer the originator. Trials were similar to the acquisition phase with the following modifications: the color of the produced square was either congruent (i.e., the same color that a particular response produced in the acquisition phase) or incongruent (i.e., the color that was previously produced by the other response). Moreover, the delay between action and outcome was manipulated so that the colored square appeared on the screen either immediately after the response was given or at a delay of either 300 or 600 ms. Finally, after the colored square disappeared from the screen, participants were asked to rate their perceived agency over the action-outcome (i.e., their certainty that they had caused the square to appear on the screen). To this end, a Likert scale was presented and participants indicated their answers on a scale from 1 to 4, with one representing absolute certainty that the computer had produced the square and four representing absolute certainty that the square was produced by oneself. Ratings were given with the left hand fingers using the buttons ‘Z,’ ‘X,’ ‘C,’ and ‘V’ of a QWERTY keyboard. Importantly, to avoid a contamination of the ratings by response biases, two different rating scales were presented across trials (randomly intermixed). These two scales were of opposite polarity (i.e., starting from 1 on the left to 4 on the right or vice versa). Prior to starting the test phase, participants were familiarized with the general task and the rating procedure. They first performed ten practice trials in which only the rating scales were presented followed by another ten practice trials with the complete task, supervised by the experimenter.

For both experimental phases, we first computed the proportion of left and right hand responses and the mean response times (RTs). Both scores were compared between the control session and the anti-free will session via paired-samples *t*-tests (two-tailed). Agency ratings of the test phase were analyzed in a GLM using the within-subjects factors CONGRUENCY (congruent vs. incongruent), DELAY (0 vs. 300 vs. 600 ms), and SESSION (Control vs. Anti-free will).

## RESULTS

### BELIEF MANIPULATION

The comparison of participants’ FAD+ scores between the control session and the anti-free will session confirmed that participants reported stronger determinist beliefs in the anti-free will session than in the control session, *t*_50_ = 2.885, *p* = 0.003, Cohen’s *d* = 0.211 (Control: *M* = 2.88, SD = 0.64; Anti-free will: *M* = 3.01, SD = 0.59). Moreover, the aforementioned screening procedure of individual difference scores (see Meterials and Methods section) identified eleven reactant participants who reported stronger free will beliefs in the anti-free will session than in the control session. A *post hoc* ANOVA on participants’ FAD+ scores, with SESSION as a within-subjects factor and GROUP (Responder vs. Reactant) as a between-subjects factor, revealed a significant interaction such that responders exhibited stronger free will beliefs in the control session than in the anti-free will session, whereas the opposite pattern was found for reactant participants, *F*_1,49_ = 34.414, *p* < 0.001, *d* = 2.366 (Responders: Control Session *M* = 2.761, SD = 0.095; Anti-free will Session *M* = 2.986, SD = 0.094; Reactants: Control Session *M* = 3.325, SD = 0.181; Anti-free will Session *M* = 3.078, SD = 0.180).

### INTENTIONAL BINDING TASK

**FIGURE 2:**
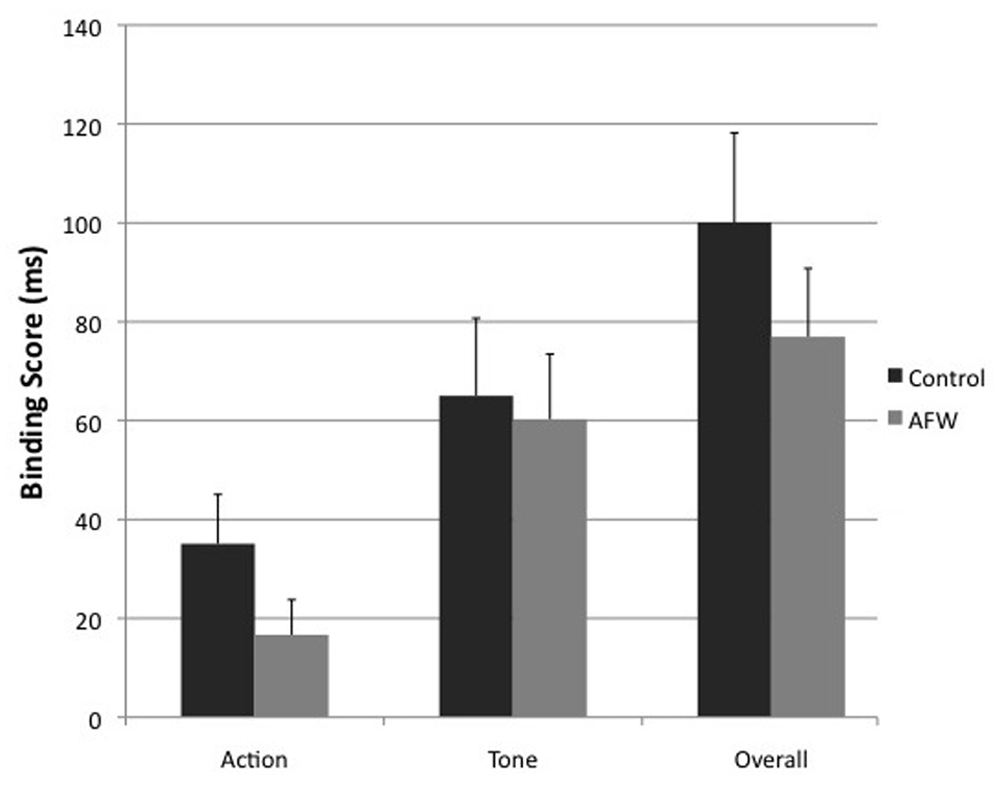
**Results of the intentional binding task.** Bars display mean binding scores and SEs for actions, tones and composite scores of both, separately for control and anti-free will conditions.

The GLM revealed a significant main effect of AGENCY, *F*_1,50_ = 6.28, *p* = 0.015, and a marginally significant trend of JUDGMENT, *F*_1,50_ = 3.032, *p* = 0.088. In addition, the interaction between JUDGMENT and AGENCY was significant, *F*_1,50_ = 36.874, *p* < 0.001, indicating that actions were perceived as occurring later in the agency blocks than in the baseline blocks (Judgment errors: Baseline *M* = -0.184 ms, SE = 6.899; Agency *M* = 25.701 ms, SE = 8.578), whereas the opposite was observed with tone judgments (Baseline *M* = 58.217 ms, SE = 7.892; Agency *M* = -4.448 ms, SE = 11.922). This pattern reflects a replication of the general intentional binding effect (Haggard et al., 2002a). Most importantly, the three-way interaction between JUDGEMENT, AGENCY, and SESSION was at trend-level, *F*_1,50_ = 2.863, *p* = 0.097, and reached significance in a one-tailed *t*-test between the intentional binding scores of the control session (*M* = 100.116 ms, SE = 18.104) and the anti-free will session (*M* = 76.973 ms, SE = 13.822), *t*_50_ = 1.692; *p* = 0.048, *d* = 0.248. This one-tailed test can be justified by the directional expectation that weakening free will beliefs would diminish the sense of agency (see **Figure 2**). All other main effects and interaction terms were non-significant (all *p* > 0.39).

**FIGURE 3:**
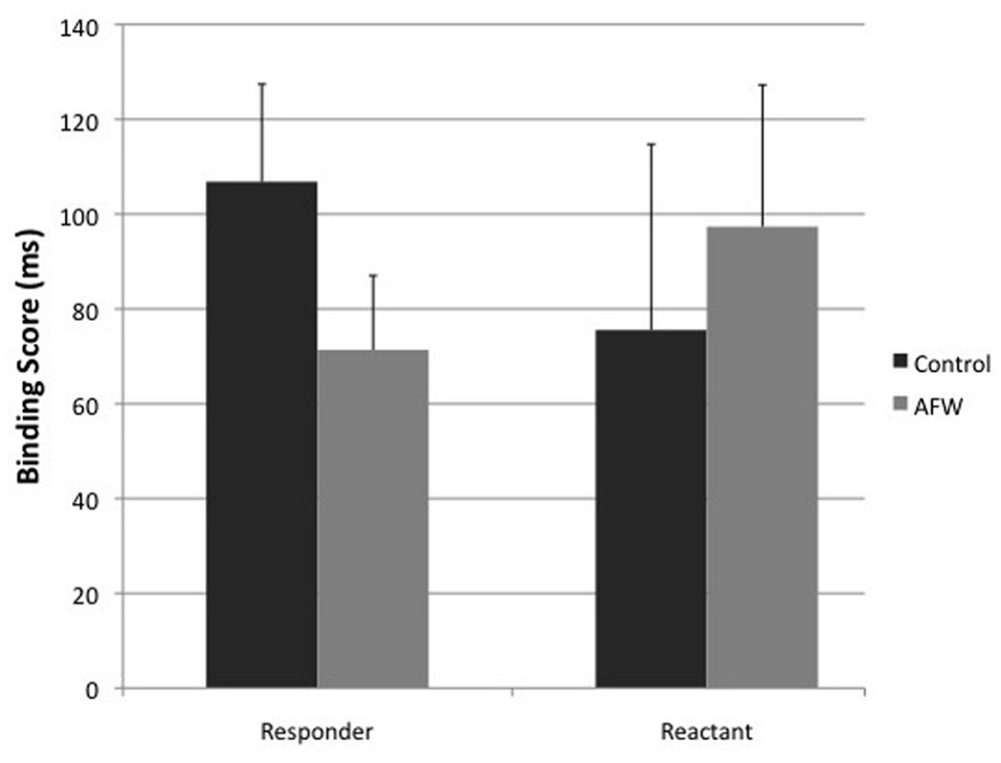
**Intentional binding composite scores for reactant and responder participants, separately for control and AFW sessions.** Error bars represent SEs.

Thereafter, we conducted explorative *post hoc* analyses in which intentional binding scores were analyzed separately for responders and reactant participants (see methods section). In the group of responders, the intentional binding scores were significantly decreased in the anti-free will session (Control session *M* = 106.866 ms, SE = 21.202; Anti-free will session *M* = 71.360 ms, SE = 15.065), *t*_39_ = 2.260; *p* = 0.015, *d* = 0.282. By contrast, in the reactant group, intentional binding scores did not differ and were in fact numerically reversed, with the anti-free will session showing greater binding scores (Control session *M* = 75.569 ms, SE = 33.856; Anti-free will session *M* = 97.381 ms, SE = 34.015), *t*_10_ = 0.909; *p* = 0.193, *d* = 0.093 (see **Figure 3**). Thus, although only of exploratory value, this analysis indicates that the overall effect of the belief manipulation on intentional binding was related to the individual effectiveness of the belief manipulation.

### SATO TASK: ACQUISITION PHASE

The analysis of participants’ RTs and response choices revealed that performance in the acquisition phase did not differ between the control session and the anti-free will session (RT: Control = 293.2 ms; Anti-free will = 299.9 ms; percentage of left and right responses: Control = 49.6 vs. 50.4%; Anti-free will = 51.8 vs. 48.2%; all *p* > 0.05).

### SATO TASK: TEST PHASE

**FIGURE 4:**
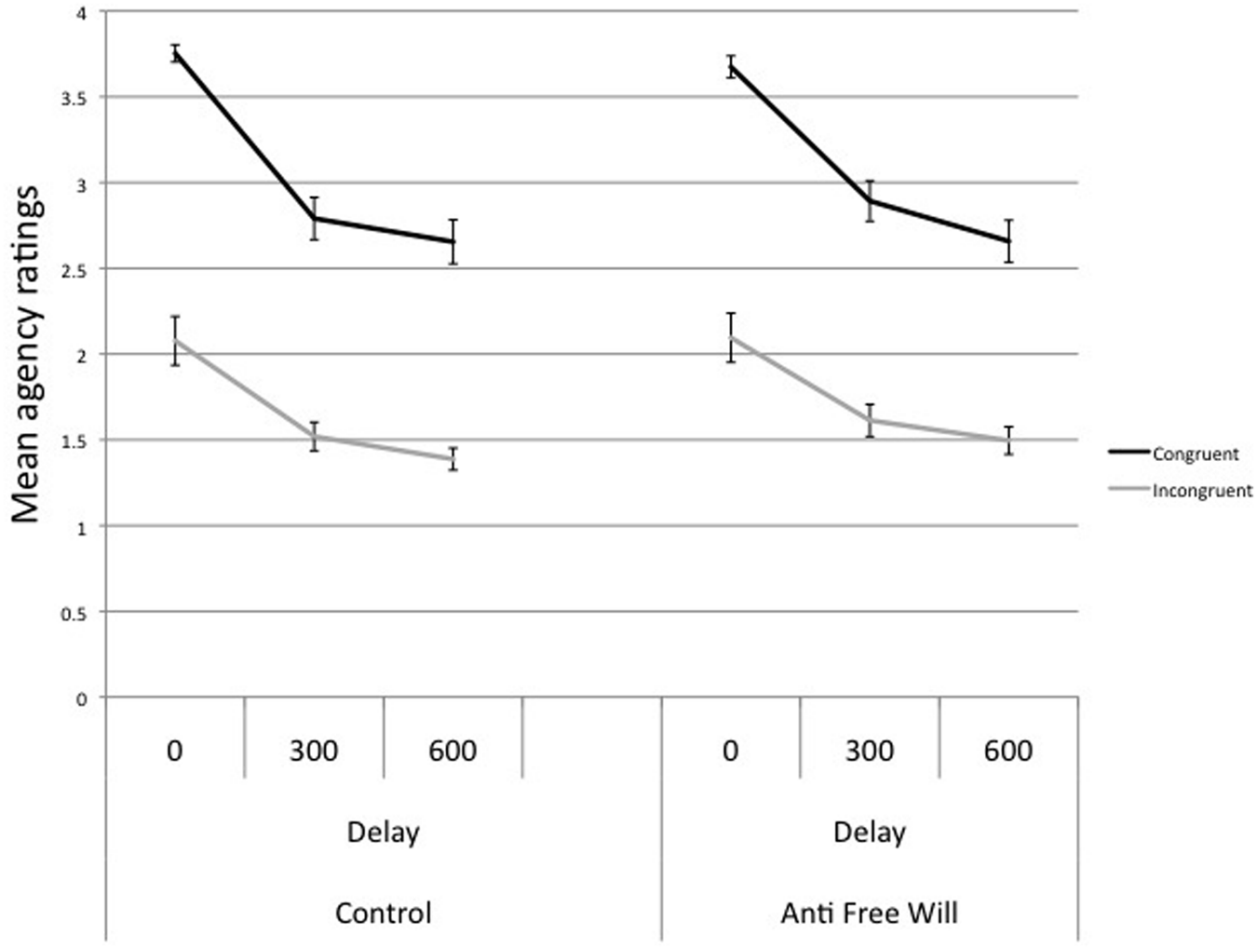
**Results of the Sato task.** Values indicate mean agency ratings as a function of congruency, delay, and belief conditions.

As in the acquisition phase, the proportion of response choices and the RTs did not differ between the two sessions (RT: Control = 320.1 ms; Anti-free will = 326.6 ms; percentage of left and right responses: Control = 52.8 vs. 47.2%; Anti-free will = 50.4 vs. 49.6%; all *p* > 0.05). Moreover, the analysis of participants’ agency ratings revealed a significant main effect of CONGRUENCY, *F*_1,50_ = 96.692, *p*< 0.001, indicating higher agency ratings on congruent trials than on incongruent trials (Congruent *M* = 3.070, SE = 0.081; Incongruent *M* = 1.698, SE = 0.080). The main effect of DELAY was significant as well, *F*_1,50_ = 48.022, *p* < 0.001, reflecting a progressive decrease of agency rating with the length of the delay (0 ms delay, *M* = 2.900, SE = 0.069; 300 ms delay *M* = 2.204, SE = 0.067; 600 ms delay *M* = 2.049, SE = 0.065). Both effects are replications of previous studies on explicit agency components (e.g., Sato and Yasuda, 2005; Spengler et al., 2009). In addition, there was a significant interaction between CONGRUENCY and DELAY, *F*_1,50_ = 16.605, *p* < 0.001, reflecting a stronger delay effect for congruent effects than for incongruent effects (see **Figure 4**). Importantly, neither the main effect of SESSION was significant, *F*_1,50_ = 1.118, *p* = 0.295, nor any interaction term involving this factor (all *p* > 0.203). Thus, our results replicated previous observations that congruency with prior action-effect contingencies, along with as the delay of the action-outcome, affects the explicit components of agency (Sato and Yasuda, 2005; Spengler et al., 2009). By contrast, agency ratings were not affected by the manipulation of free will beliefs.

As in the intentional binding section, we next conducted the *post hoc* analyses in which the data of responders and reactants were analyzed separately. In both groups, however, the same pattern was evident as in the main analysis (i.e., significant main effects of CONGRUENCY, DELAY, and a significant interaction between the two factors, but non-significant main effects of SESSION and interaction terms involving this factor). Thus, contrary to the intentional binding data, explicit agency ratings were not influenced by the effectiveness of the belief manipulation.

### COMPARISON BETWEEN IMPLICIT AND EXPLICIT AGENCY

In a final analysis aimed at directly comparing the effects of the belief manipulation on explicit and implicit components, we conducted an ANOVA with factors TASK (Intentional Binding vs. Sato) and SESSION. As the two tasks have quite different metrics, we Z-normalized both the overall intentional binding score from each session (intentional binding task) and the average agency rating across all conditions (Sato task). This ANOVA revealed non-significant main effects of TASK (due to the normalization) and SESSION, *F*_1,50_ = 0.144, *p* = 0.706, but a significant interaction between the two factors, *F*_1,50_ = 4.985, *p* = 0.030, confirming that implicit and explicit components of agency were differentially affected by the manipulation.

## DISCUSSION

The aim of the present study was to probe whether weakening belief in the concept of free will would have a causal impact on participants’ sense of agency. In line with our hypothesis, we found that intentional binding was significantly reduced in the anti-free will condition, indicating that determinist beliefs hamper the implicit sensation of being in control of one’s actions. By contrast, participants’ explicit agency ratings were not affected by the belief manipulation.

### FREE WILL BELIEFS AND SELF-CONTROL

The present study complements and extends a growing body of research on the interaction between high-level determinist beliefs and low-level processes involved in self-control. Previous studies have shown that participants induced to disbelieve in free will exhibit less involvement in motor preparation (Rigoni et al., 2011), do not adapt their behavior in response to unwanted action-outcomes (Rigoni et al., 2013), and are less inclined to engage in effortful cancellation of prepotent behavior (Rigoni et al., 2012; Lynn et al., 2013). Our findings add several new and valuable insights to this existing literature. First and foremost, establishing a causal link between determinist beliefs and the sense of agency provides an integrative mechanism that explains how free will beliefs impact behavioral control in a variety of contexts. It is possible that free will beliefs are able to influence the entire action cycle by intervening at an early or pre-reflective level. This impact on sensorimotor binding then cascades to more overt behavior, eliciting in turn less intentional effort, a reduced sense of agency, and less feeling of responsibility. Interestingly, a recent study has linked the intentional binding effect to the feeling of responsibility (Moretto et al., 2011). The authors employed a modified intentional binding paradigm in which actions had unpredictable consequences, either in a moral context or a simple economic context. Intentional binding was enhanced when actions were embedded in a moral context, suggesting binding is sensitive to a feeling of responsibility over self-produced action consequences. It is tempting to speculate that an intrinsic bias to bind actions with their outcomes in time could constitute a building block of higher-order social cognition, and in light of these findings it becomes more apparent why the manipulation of free will beliefs can impact such a wide range of behaviors.

Finally, from a methodological perspective, our study is the first to employ a successful within-subjects manipulation of free will beliefs. As such, it provides more direct evidence for the causal impact of high-level beliefs, since pre-existing differences between different groups of participants can be ruled out as an alternative explanation. Moreover, having established the feasibility of this manipulation in within-subjects designs may permit its application in new experimental settings, e.g., in combination with brain imaging techniques such as fMRI.

### IMPLICIT AND EXPLICIT COMPONENTS OF AGENCY

Beyond establishing a causal link between free will beliefs and agency, our data revealed marked differences between implicit and explicit components of agency. While intentional binding scores were reduced in the anti-free will session, the explicit agency ratings in the Sato task were not affected. This observation is interesting for several reasons. First, it indicates that the influence of free will beliefs on agency does not extend to the level of reflective deliberation. Instead, beliefs seem to bias very basic and implicit processes that underlie our pre-reflective self-perception as intentional agents. In cases where the integration of agency cues into judgment is transparent, explicit processes may override the modulation of pre-reflective prediction processes. In addition, the dissociation between implicit and explicit agency components also speaks against the possibility that our data may originate from “demand effects” (i.e., participants form expectations about the purpose of the experiment and try to please the experimenter by fulfilling his/her hypotheses). Finally, on a theoretical level, this result corroborates the notion that the sense of agency is not a unitary psychological concept, but rather entails distinct components that tap into different facets of action awareness (see David et al., 2008; Synofzik et al., 2008; Obhi and Hall, 2011). In line with this view, a recent study indicated that explicit and implicit components might operate at different time scales. Ebert and Wegner (2010) employed a paradigm that simultaneously assessed implicit and explicit agency components in a relatively naturalistic task setting. Participants were instructed to push or pull a lever, causing objects on a screen to either come closer or move further away from them. As in the present study, the authors manipulated the congruency between actions and outcomes, and the outcome delay. It was found that both measures were influenced by action-outcome congruency (i.e., both implicit and explicit agency was higher for congruent outcomes). However, the influence of congruency was more pronounced for explicit agency, particularly with long outcome delays, suggesting that implicit components may operate on a shorter time scale.

### LIMITATIONS AND OUTLOOK

Despite the clear overall patterns of results, several aspects of our data indicate the need for further investigation. First, it must be considered that the effect of the belief manipulation on IB reached significance only in a one-tailed *t*-test. The *post hoc* analyses indicated that this was likely due to the variability in participants’ responses to the manipulation: There was a robust attenuation of intentional binding in the group of responders, but in the group of reactant participants this effect was not only absent but even reversed numerically. On the one hand, this finding is reassuring, as it confirms that the effect is related to the effectiveness of the belief manipulation. On the other hand, it raises new questions, in particular what determines an individual’s response, and whether a reactant response only eliminates the effects of the free will manipulation or if it can even increase agency as compared to a control session. The rather small number of reactant subjects in our sample prohibits us from making conclusions about this issue. Furthermore, the precise nature of the implicit processes responding to free will beliefs remains to be uncovered through future research. While intentional binding has primarily been related to motor prediction, the findings of Aarts and van den Bos (2011) indicate that non-motor predictive and inferential processes might be affected as well. Future studies should also attempt to minimize confounds of the predominant free-will belief manipulation (e.g., differences in the length or the emotionality of the anti-free will text and the control text) so that findings can be more unequivocally attributed to the strength of free will beliefs.

## CONCLUSION

The present study revealed that inducing disbelief in the concept of free will has a causal impact on implicit components of agency, but not on explicit agency components. This finding points to a psychological mechanism through which determinist beliefs exert their wide-ranging influence on human behavior, and highlights the multi-dimensional nature of the sense of agency.

## Conflict of Interest Statement

The authors declare that the research was conducted in the absence of any commercial or financial relationships that could be construed as a potential conflict of interest.

## Footnotes

1 We employed one-tailed significance tests in all comparisons for which we had clear directional expectations. This is the case for the comparison of intentional binding scores or agency ratings between the AFW session and control session (where we expected the AFW manipulation to decrease participants’ agency). However, this is not the case for undirected comparisons (e.g., the test between the frequency of left- and right-hand responses in the acquisition phase of the Sato task).
